# Mental disorders, attrition at follow‐up, and questionnaire non‐completion in epidemiologic research. Illustrations from the CoLaus|PsyCoLaus study

**DOI:** 10.1002/mpr.1805

**Published:** 2019-09-30

**Authors:** Marc Dupuis, Marie‐Pierre F. Strippoli, Mehdi Gholam‐Rezaee, Martin Preisig, Caroline L. Vandeleur

**Affiliations:** ^1^ Institute of Global Health, Department of Medicine University of Geneva Geneva Switzerland; ^2^ Centre for Research in Psychiatric Epidemiology and Psychopathology, Department of Psychiatry Lausanne University Hospital Lausanne Switzerland

**Keywords:** attrition, CoLaus|PsyCoLaus study, mental disorders, non‐response, self‐rating questionnaires

## Abstract

**Objective:**

This study aimed to investigate the associations between mental disorders recorded at baseline and participation in the subsequent follow‐up interview (vs. attrition) or baseline questionnaire completion (vs. non‐response) within the psychiatric arm of a population‐based study.

**Methods:**

Participants of a physical health survey were initially invited to also participate in a semi‐structured interview covering mental disorders and were reassessed approximately 5.5 years later. They were also asked to complete self‐rating questionnaires at baseline. Associations between the presence of lifetime mental disorders assessed at baseline and attrition at follow‐up as well as non‐completion of self‐rating questionnaires at baseline were established.

**Results:**

After controlling for sociodemographic variables, a significant negative association was found between anxiety disorders at baseline and attrition at follow‐up (Adjusted odds ratio (AOR) *=* 0.84; 95% confidence interval (CI) = 0.71–1.00) and a positive association between major depressive disorders (MDD) and non‐response to the self‐rating questionnaires at baseline (AOR *=* 1.24; 95% CI = 1.05–1.45).

**Conclusions:**

The associations of anxiety disorders during lifetime with a higher participation rate in interviews at follow‐up and of MDD during lifetime with the non‐completion of self‐rating questionnaires are potential sources of bias and should be taken into account in future longitudinal research.

## 1 INTRODUCTION

Non‐response is a major issue in both epidemiological studies and sociological surveys. High response rates tend to alleviate bias (Groves & Peytcheva, 2008), whereas low response rates make study validity questionable. Though low response rates do not automatically induce bias (Nohr, Frydenberg, Henriksen, & Olsen, 2006), non‐response bias must always be suspected in such cases. Longitudinal designs are suited for multiple purposes, but they make this issue even more serious by multiplying losses across the successive stages of a study (Howe, Tilling, Galobardes, & Lawlor, 2013). Non‐response at follow‐up stages of studies becomes the epidemiologist's nightmare that we call “attrition” (Deeg, 2002).

As pointed out by several authors (Galea & Tracy, 2007; Studer et al., 2013; Tolonen et al., 2006; Tourangeau & Yan, 2007; Zhao, Stockwell, & Macdonald, 2009), non‐response rates in survey research have grown drastically. In health studies in particular, health issues can represent a major factor of both non‐response and attrition, making the groups of interest underestimated and less representative and also lowering the statistical power of the analyses (de Graaf, Bijl, Smit, Ravelli, & Vollebergh, 2000; Goldberg, Chastang, Zins, Niedhammer, & Leclerc, 2006).

In psychiatric epidemiology, this issue is of crucial importance. Despite the fact that systematic research on the question remains scarce, several studies have shown that mental disorders have a significant association with non‐response and attrition (Badawi, Eaton, Myllyluoma, Weimer, & Gallo, 1999; Chatfield, Brayne, & Matthews, 2005; de Graaf et al., 2000; de Winter et al., 2005; Dupuis et al., 2014; Dupuis, Baggio, Mohler‐Kuo, & Gmel, 2015; Eaton, Anthony, Tepper, & Dryman, 1992; Gnambs & Kaspar, 2016; Hill, Roberts, Ewings, & Gunnell, 1997; Korkeila et al., 2001; Saiepour et al., 2019; Studer et al., 2013; Suominen et al., 2012; Tambs et al., 2009; Van der Veen, Van der Meer, & Penninx, 2009; Wolke et al., 2009). In contrast, even if cumulated diagnoses of mental disorders imply a higher risk for non‐participation in a given study (Allott, Chanen, & Yuen, 2006; de Graaf et al., 2000; Eaton et al., 1992; Saiepour et al., 2019), healthy subjects generally feel less concerned by epidemiologic studies, which entails an even higher risk to drop out. For example, individuals who do not drink at all are more likely to refuse participation in studies regarding alcohol use than moderate drinkers (Dupuis et al., 2014). Finally, systematic psychiatric studies are particularly likely to cover sensitive questions (e.g., concerning drug use, past traumatic experiences, sexual life, etc.) that are well‐known to cause both specific and overall non‐response and attrition as well (Gnambs & Kaspar, 2015, 2016; Saiepour et al., 2019; Tourangeau & Yan, 2007; Zhao et al., 2009). Investigating factors of non‐response and attrition in epidemiologic research remains thus of ever‐growing importance.

Non‐response and attrition correspond to two different notions, even if some authors consider that they correspond to a same phenomenon taking place in different temporalities (e.g., Badawi et al., 1999). Although the former refers to unreached participants in cross‐sectional study designs, the latter refers to participants lost at follow‐up in longitudinal studies. Both may lead to study bias: selection or non‐response bias and attrition bias, respectively. Non‐response bias mainly impacts study representativeness, even if multiple factors impacting non‐response may also distort between‐measures associations and between‐group differences. Attrition bias leads to an even higher risk of causing such biased results, including making the population study results less representative.

Both bias have however also much in common. Another difference is the fact that, when talking about non‐response, researchers have very little information about the unreached participants (which is exceptionally not the case in this study). Despite that each of those bias can be related to specific factors, both can be caused by common factors. For instance, factors that have a role on non‐response can repeatedly impact participation at follow‐up, reinforcing their own biasing effect under the form of attrition bias. Though the common factors represent an important threat to be addressed, little has been undertaken so far to investigate potential differences in attrition and non‐response bias linked with different data collection methods (i.e., face‐to‐face interviews vs. questionnaires). Indeed, most of studies covering the impact of mental health issue on participation are based on questionnaires, making the effect of mental health on participation in interviews less explored so far. In other words, the potential benefits of each method in order to prevent studies from specific bias remain unknown. The main aim of this paper was thus to study the associations between mental disorders established at the baseline assessment (i.e., their presence during lifetime as a proxy for both stable and episodic mental disorders; for instance, alcohol and drug use disorders and mood disorders, respectively) and subsequent participation in diagnostic interviews and with self‐rating questionnaire completion at the baseline assessment in a population‐based study.

## 2 METHODS

### 2.1 Study design, recruitment procedure, information, and consent

The data of the present paper stemmed from CoLaus|PsyCoLaus (Firmann et al., 2008; Preisig et al., 2009), a prospective cohort study designed to study mental disorders and cardiovascular risk factors in the community and to determine their associations. The cohort was randomly selected from the 35‐ to 75‐year‐old residents of the city of Lausanne (Switzerland) from 2003 to 2006 according to the civil register. A total of 6,733 subjects accepted the physical baseline evaluation. Sixty‐seven percent of participants between 35 and 66 years of age (*n* = 5,535) also accepted the psychiatric evaluation, resulting in a sample of 3,719 individuals.

All participants of the baseline assessments were re‐contacted approximately 5 years later to take part in a follow‐up evaluation. Among the participants of the psychiatric baseline evaluation, which took part at the university hospital, 2,852 (76.5%) agreed again to participate, whereas 51 had died, 87 had migrated, 535 refused participation, and 203 could not be reached.

The Institutional Ethics' Committee of the University of Lausanne approved the CoLaus|PsyCoLaus study. All participants signed a written informed consent after having received a detailed description of the goal and funding of the study. They could leave the study and refuse to answer questions at any time, in accordance with the Helsinki Declaration.

In addition to the semi‐structured interview, about 30 pages of self‐rating questionnaires assessing several psychological and familial aspects were systematically distributed to the participants at baseline, which 2,704 (72.5%) completed and sent back by post. The questionnaires completed at baseline are listed in Appendix A.

### 2.2 Measurements

At the baseline and follow‐up evaluations, eligible persons first received an information letter. A member of the research team then contacted them by phone in order to schedule an appointment. For the psychiatric evaluation, the interviews could either take place at different localizations of the university hospital or at the participants' homes, depending on their preference. In addition to the semi‐structured interview, the self‐rating questionnaires assessing several psychological and familial characteristics were systematically distributed to the participants during the psychiatric baseline evaluations.

Age was recorded when participants were recruited. Information on mental disorders at baseline was collected using the French version (Leboyer et al., 1995; Preisig, Fenton, Matthey, Berney, & Ferrero, 1999) of the Diagnostic Interview for Genetic Studies (DIGS; Nurnberger et al., 1994). The DIGS is a semi‐structured interview that elicits information on specific symptoms for the major Axis‐1 mental disorders as defined in the DSM‐IV‐TR (American Psychiatric Association, 2000). The French version revealed excellent interrater agreement and slightly lower test–retest reliability for psychotic mood disorders (Preisig et al., 1999) and substance use disorders (Berney, Preisig, Matthey, Ferrero, & Fenton, 2002). As the original DIGS did not include questions assessing generalized anxiety disorder and post‐traumatic stress disorder, the corresponding modules of the Schedule for Affective Disorders and Schizophrenia—Lifetime and Anxiety disorder version (SADS‐LA; Endicott & Spitzer, 1978; Mannuzza, Fyer, Klein, & Endicott, 1986) were added. Similarly, the brief phobia chapter of the DIGS was replaced by the more extensive section of the SADS‐LA. Except for generalized anxiety disorder, we documented fair to good interrater and test–retest reliability for specific anxiety disorders (Rougemont‐Buecking et al., 2008).

At follow‐up, an interview covering any new occurrence of mental disorders or episodes since the baseline interview was conducted using a shortened version of the DIGS. The interviews were conducted by master's level psychologists who had been trained for a period of 1 to 2 months. Each interview and diagnostic assignment was reviewed by a senior psychologist.

The diagnostic assignment was based on the DSM‐IV criteria (American Psychiatric Association, 2000). Main Axis‐1 disorders were grouped as follows: episodes of major depressive disorder (MDD) and bipolar disorder (bipolar‐I and bipolar ‐II disorders), anxiety disorders (agoraphobia, panic disorder, generalized anxiety disorder, and social phobia), psychotic disorders (schizophrenia, schizoaffective disorders, schizophreniform disorder, and brief psychotic disorder), alcohol use disorders (alcohol abuse and dependence), and drug use disorders (drug abuse and dependence). For every statistical analysis, the presence of each mental disorder during lifetime was established using baseline information. Socioeconomic status (SES) was assessed at baseline using Hollingshead's four‐factor index (Hollingshead, 1975).

### 2.3 Statistical analyses

First, subjects were grouped into two categories depending on their participation: *regular participants* (i.e., who took part in both the baseline and follow‐up studies) and *attritors* (i.e., who participated in the baseline study but did not participate in the follow‐up). Between‐group analyses were performed using chi‐square tests or analysis of variance as appropriate. Then, the presence of mental disorders during lifetime measured at baseline were used as the predictors of attrition at follow‐up. Next, lifetime mental disorders assessed at the baseline interview were linked to non‐completion of the self‐rating questionnaires at baseline. Logistic regression models were first applied (resulting in crude odds ratios: ORs) for each unadjusted mental disorder separately. Then, regression models covering all groups of mental disorders together, adjusted for gender, nationality (Swiss vs. others), mother tongue (French vs. others), age, and SES at baseline were applied (resulting in adjusted odds ratios: AORs) by introducing all the variables into one overall model. The statistical analyses were run using SPSS 25.

## 3 RESULTS

### 3.1 Descriptive statistics

A total of 3,728 participants (mean age = 50.99 ± 8.83) took part at baseline (Table 1). There were 2,852 participants who completed both baseline and follow‐up interviews (regular participants) and 876 subjects who completed only the baseline interview (attritors). The participants included 1,755 (47.1%) men and 1,973 (52.9%) women; 2,629 (70.5%) Swiss citizens and 1,099 (29.5%) participants from other countries; 2,357 (63.2%) individuals whose mother tongue was French and 1,371 (36.8%) individuals whose mother tongue was another language (i.e., mostly German or Italian, which are two other official Swiss languages, or Spanish and Portuguese).

**Table 1:** Sociodemographic variables and mental disorders at baseline as a function of type of participation

Variable	Total	Regular participants (BL and FU)	Attritors (only BL)
*N*	3,728	2,852	876
Age at baseline			
35 to 40 years	458	12.2%	12.4%
40 to 45 years	696	18.7%	18.6%
45 to 50 years	686	18.8%	17.0%
50 to 55 years	597	15.8%	16.6%
55 to 60 years	518	13.9%	13.9%
60 to 65 years	552	14.8%	13.8%
65 years and older	231	5.8%	7.6%
Gender			
Male	1,755	46.2%	50.1%
Female	1,973	53.8%	49.9%
SES			
Low	407	10.0%	13.9%
Low to medium	468	12.0%	14.4%
Medium	1,087	28.1%	32.8%
Medium to high	860	23.8%	20.8%
High	906	26.2%	18.2%
Nationality			
Swiss	2,629	73.8%	59.2%
Others	1,099	26.2%	40.2%
Mother tongue			
French	2,357	65.9%	54.6%
Others	1,371	34.1%	45.4%
Major depressive disorder^a^			
Present	1,624	43.8%	43.2%
Absent	2,098	56.2%	56.8%
Bipolar disorder^a^			
Present	69	1.9%	1.8%
Absent	3,646	98.1%	98.2%
Anxiety disorders^a^			
Present	1,262	35.3%	30.3%
Absent	2,432	64.7%	69.7%
Psychotic disorders^a^			
Present	23	0.6%	0.6%
Absent	3,687	99.4%	99.4%
Alcohol use disorders^a^			
Present	438	11.2%	13.7%
Absent	3,271	88.8%	86.3%
Drug use disorders^a^			
Present	230	6.3%	6.0%
Absent	3,480	93.7%	94.0%

Abbreviations: BL, baseline; FU, follow‐up; *M*, means; SES, socioeconomic status; *SD*, standard deviation.

a At least one diagnosis was missing for several participants (*N* = 6 to 34).

### 3.2 Participation in the interview‐based parts of the study

As reported in Table 2, significant unadjusted associations were found between attrition and each sociodemographic variable, except age. In particular, a lower risk of attrition was found among Swiss‐ and French‐speaking participants (OR = 0.53, *p* < .001, and OR *=* 0.62, *p* < .001, respectively). In addition, participants from the highest SES groups were also at lower risk of attrition (OR_Medium to high vs. low_ = 0.63, *p* = .001, and OR_High vs. low_ *=* 0.50, *p* < .001, respectively). Concerning mental disorders, the only significant difference measured in terms of the presence of disorders at baseline between participant groups concerned anxiety disorders. The participants having suffered from at least one anxiety disorder were less likely to drop out (OR = 0.80, *p* = .007). When controlling for sociodemographic variables and every other disorder in an overall model, this association remained similar (AOR = 0.84, *p* = .050). The association between attrition at follow‐up and nationality also reached statistical significance in the overall model (AOR = 0.61, *p* < .001). The difference in terms of attrition between low‐ and high‐SES categories remained significant (AOR = 0.60, *p* < .001); this was however not the case for the difference between low‐SES participants and those with a medium‐to‐high SES (AOR = 0.76, *p* < .055).

**Table 2:** Unadjusted and adjusted logistic regression models assessing non‐completion (vs. completion) of follow‐up interviews as a function of socio‐demographics and mental disorders at baseline^a^

Variable	Unadjusted				Adjusted (*N =* 3,694)		
	*N*	OR	95% CI	*p*	OR	95% CI	*p*
Age at baseline	3,728						
35 to 40 years		1.00			1.00		
40 to 45 years		0.98	0.74–1.23	.882	0.93	0.70–1.23	.591
45 to 50 years		0.88	0.67–1.18	.410	0.90	0.67–1.21	.488
50 to 55 years		1.03	0.77–1.37	.854	1.08	0.80–1.44	.631
55 to 60 years		0.99	0.73–1.33	.928	1.05	0.78–1.43	.737
60 to 65 years		0.92	0.69–1.24	.581	1.01	0.74–1.37	.951
65 years and older		1.31	0.92–1.87	.140	1.43	0.99–2.07	.058
Gender (female vs. male)	3,728	**0.85**	**0.73–0.99**	**.040**	**0.84**	**0.72–0.98**	**.028**
SES	3,728						
Low		1.00			1.00		
Low to medium		0.86	0.64–1.16	.318	0.91	0.67–1.23	.910
Medium		0.84	0.65–1.08	.168	0.96	0.74–1.24	.739
Medium to high		**0.63**	**0.48–0.82**	**.001**	0.76	0.58–1.01	.055
High		**0.50**	**0.38–0.65**	**<.001**	**0.60**	**0.48–0.79**	**<.001**
Nationality (Swiss vs. others)	3,728	**0.53**	**0.45–0.62**	**<.001**	**0.61**	**0.50–0.75**	**<.001**
Mother tongue (French vs. others)	3,728	**0.62**	**0.53–0.73**	**<.001**	0.83	0.69–1.01	.056
Major depressive disorder	3,722	0.98	0.84–1.14	.753	1.04	0.88–1.23	.634
Bipolar disorder	3,715	0.99	0.56–1.74	.972	0.89	0.48–1.68	.740
Anxiety disorders	3,694	**0.80**	**0.68–0.94**	**.007**	**0.84**	**0.71–1.00**	**.050**
Psychotic disorders	3,710	0.92	0.34–2.47	.862	0.70	0.23–2.13	.534
Alcohol use disorders	3,709	1.25	0.98–1.57	.053	1.21	0.94–1.54	.137
Drug use disorders	3,710	0.96	0.70–1.32	.801	0.91	0.65–1.29	.613

Abbreviations: CI, confidence interval; OR, odd ratio; SES, socioeconomic status.

a Significant results are displayed in bold.

### 3.3 Participation in the questionnaire‐based substudy at baseline

The analyses comparing participants who completed the baseline questionnaire with those who did not complete it resulted in two significant findings (Table 3). Indeed, significant unadjusted associations were measured between each sociodemographic variable and participation in self‐rating questionnaires. Consistent with the results covering participation in follow‐up interviews, the largest differences were found between Swiss and foreign participants (OR *=* 0.46, *p < .001*) and French‐ and non‐French‐speaking participants (OR *=* 0.43, *p < .001*). Noteworthy, significant differences in terms of non‐responses were found between participants aged between 35 and 40 years and every group of age, except people aged between 40 and 45 years. The differences were even larger with people aged above 60 years (ORs *<* 0.50). In addition, a significant difference concerned the lifetime presence of drug use disorders (OR = 1.52, *p* = .003) in the corresponding unadjusted model; nevertheless, the effect was no longer significant when controlling for the sociodemographic variables. When taking all variables into account in the overall model, significant ORs ranging from 0.48 to 0.74 were found concerning sociodemographic variables. The largest effect concerned the difference between the youngest and the oldest groups of participants (AOR = 0.48). SES was however not significantly associated to non‐response when controlling for other variables. Regarding mental disorders, a significant association was found, however, only after controlling for potential confounders, between a history of MDD and non‐response to the questionnaire at baseline (AOR = 1.24, *p* = .009).

**Table 3:** Unadjusted and adjusted logistic regression models assessing non‐completion (vs. completion) of the baseline questionnaires as a function of socio‐demographics and mental disorders at baseline^a^

Variable	Unadjusted				Adjusted (*N =* 3,694)		
	*N*	OR	95% CI	*p*	OR	95% CI	*p*
Age at baseline	3,728						
35 to 40 years		1.00			1.00		
40 to 45 years		1.05	0.82–1.35	.676	1.00	0.78–1.29	.999
45 to 50 years		**0.74**	**0.58–0.96**	**.021**	**0.74**	**0.57–0.96**	**.023**
50 to 55 years		**0.63**	**0.50–0.82**	**.001**	**0.64**	**0.48–0.84**	**.001**
55 to 60 years		**0.58**	**0.44–0.77**	**<.001**	**0.62**	**0.47–0.83**	**.001**
60 to 65 years		**0.45**	**0.69–1.24**	**<.001**	**0.49**	**0.36–0.66**	**<.001**
65 years and older		**0.47**	**0.92–1.87**	**<.001**	**0.48**	**0.33–0.71**	**<.001**
Gender (female vs. male)	3,728	**0.73**	**0.63–0.84**	**<.001**	**0.74**	**0.64–0.86**	**<.001**
SES	3,728						
Low		1.00			1.00		
Low to medium		0.91	0.68–1.21	.506	0.90	0.66–1.22	.494
Medium		0.85	0.67–1.09	.207	1.04	0.80–1.35	.772
Medium to high		**0.72**	**0.55–0.93**	**.011**	0.84	0.57–1.10	.206
High		**0.64**	**0.50–0.83**	**.001**	0.76	0.63–1.00	.051
Nationality (Swiss vs. others)	3,728	**0.46**	**0.40–0.54**	**<.001**	**0.76**	**0.63–0.92**	**.004**
Mother tongue (French vs. others)	3,728	**0.43**	**0.37–0.50**	**<.001**	**0.53**	**0.45–0.64**	**<.001**
Major depressive disorder	3,722	1.15	0.99–1.33	.065	**1.24**	**1.05–1.45**	**.009**
Bipolar disorder	3,715	1.25	0.75–2.09	.386	1.25	0.71–2.20	.433
Anxiety disorders	3,694	0.92	0.80–1.08	.349	0.96	0.81–1.13	.620
Psychotic disorders	3,710	1.17	0.48–2.85	.731	1.03	0.39–2.74	.948
Alcohol use disorders	3,709	1.16	0.93–1.44	.180	1.04	0.82–1.32	.756
Drug use disorders	3,710	**1.52**	**1.15–2.01**	**.003**	1.21	0.88–1.64	.229

Abbreviations: CI, confidence interval; OR, odd ratio; SES, socioeconomic status.

a Significant results are displayed in bold.

## 4 DISCUSSION

This study aimed to investigate the associations between lifetime mental disorders assessed at baseline and attrition or/and non‐response in the CoLaus|PsyCoLaus follow‐up interview and baseline questionnaire completion. In brief, the regression models resulted in only two significant associations between mental disorders and attrition at follow‐up or non‐completion of the self‐rating questionnaires at baseline. Each part of the study resulted in one significant association with a specific group of mental disorders. On one hand, this finding highlighted the relatively small impact of mental disorders on participation to both data collections. On the other hand, this result also supports the idea of specific factors depending on the data collection method chosen. Although this study shows a negative association between anxiety disorders and attrition at follow‐up, suggesting that participants with a lifetime history of anxiety disorders at baseline were potentially less likely to drop out (AOR = 0.84, *p =* .050), it also shows that participants with a lifetime history of major depression assessed at baseline could have lacked motivation to complete the self‐rating questionnaires or to mail them back to the study team (AOR = 1.24; *p* = .009). Both results are of interest and merit to be discussed separately.

Concerning follow‐up interviews, the absence of positive associations between Axis‐1 mental disorders and attrition at follow‐up means that participation in the CoLaus|PsyCoLaus study at follow‐up was not explained by the presence of mental disorders at the baseline assessment. In fact, anxiety disorders were associated with a lower attrition rate at follow‐up, which was contrary to our expectations. Resulting from the review of literature, some evidences for positive associations between anxiety disorders and attrition were found instead. Nevertheless, most of studies consisted of questionnaire‐based surveys (Tambs et al., 2009; Van der Veen et al., 2009); other studies showed that anxiety disorders had an effect on losing contact with participants, that is to say, they lead to more silent refusal concerning interviews (Eaton et al., 1992); finally, the role of anxiety disorders on attrition was eventually not confirmed in a further investigation based on one of the aforementioned study data (Lamers et al., 2012). Concerning the present study, participants received phone calls in order to set a date for the interview and were given the possibility to be interviewed at home. Such conditions probably made individuals with anxiety disorders hardly refuse to participate in interviews. Although it is already known that anxious and depressive subjects are less able to explicitly refuse study participation and are more likely to become passive/silent refusers, mostly in surveys (Bambs et al., 2013; Dupuis et al., 2015; Eaton et al., 1992; Vega et al., 2010), very little has been undertaken so far to quantify the associations between anxiety disorders and attrition in interview‐based studies.

Regarding the self‐rating questionnaires, the association between a history of major depression and non‐response, even after controlling for potential confounders, should be noted. In addition, participants aged between 35 and 45 years were at higher risk of non‐response to the questionnaire. Such an association was probably attributable to parental responsibilities, making questionnaire completion more difficult for these age groups. Finally, drug use disorders revealed potential associations with non‐response to the questionnaires, which has previously been published (Dupuis et al., 2014; Vega et al., 2010; Zhao et al., 2009), but in our study this association did not reach statistical significance after controlling for sociodemographic confounding variables.

Both significant AORs that were respectively found for anxiety disorders and attrition, and MDD and non‐response represent relatively small effects (Chen, Cohen, & Chen, 2010). Nonetheless, the same participation bias could be repeated and thus reinforced in multiple‐stage longitudinal studies, which potentially makes their effects of relative importance for longitudinal studies. Moreover, given the weak yet still significant association between a history of MDD assessed at baseline and the non‐completion of the questionnaires, further analyses of this data should consider the potential role of the lower likelihood of MDD in the associations involving mental disorders or the cardiovascular risk with psychological or familial factors.

A last point that merits to be discussed concerns the high lifetime prevalence of MDD measured. As stated by Vandeleur et al. (2017), both Swiss population‐based studies covering depression, the CoLaus|PsyCoLaus study and the Zurich cohort study, resulted in the highest prevalence of depressive disorders (i.e., MDD and new DSM‐5 categories) reported so far. Though they were conducted in different linguistic areas using different research instruments, both studies have in common to be based on semi‐structured interviews, which could have led to higher prevalence rates than studies based on fully structured interviews (Vandeleur et al., 2017).

The CoLaus|PsyCoLaus study has several strengths including a comprehensive psychiatric assessment at baseline conducted face to face by master's level psychologists and a relatively low attrition and non‐response to questionnaire rate in a large sample. However, the present analysis also entails at least three limitations. First, attrition at follow‐up could have been associated with the presence of mental disorders at the follow‐up, but this could not be assessed as diagnostic information at follow‐up and, was by definition, lacking in attritors. Indeed, it seems plausible to assume that the presence of disorders at the follow‐up may have been more strongly associated with attrition than disorders assessed at baseline, even if the latter were assessed for the participants' entire lifetime. Second, the role of other potential confounders which could have influenced attrition were not assessed in this study. For instance, sexual orientation was not assessed despite its association with suicidality found in Switzerland few years ago (Wang et al., 2014). Given that CoLaus|PsyCoLaus is a psychiatric study, our analyses only focused on the associations between mental disorders, controlled for sociodemographic confounders, and attrition and non‐response, despite the fact that other factors certainly influenced non‐participation. In other words, as attrition was not explained by the presence of mental disorders at baseline, other factors must indeed have played a role. Finally, the presence of lifetime MDD assessed at baseline only partially accounted for non‐response to the self‐rating questionnaires, and other factors that were not assessed in this study must have influenced the non‐completion of the questionnaires.

In conclusion, the current study revealed only two weak associations between the presence of mental disorders at baseline and attrition at follow‐up and non‐response to the baseline questionnaires. Nevertheless, the CoLaus|PsyCoLaus study is the first attempt to assess the presence of mental disorders in a large sample from the Swiss French‐speaking population in the age range of 35 to 75 years. The study is of high interest given that the same participants took part in the CoLaus study, which has assessed the presence of cardiovascular risk factors and diseases. This study therefore contributes to the scientific knowledge on the relations between cardiovascular risk factors or diseases and mental disorders. Finally, the CoLaus|PsyCoLaus study was completed by self‐rating questionnaires, although non‐response was weakly but still significantly associated with a lifetime history of MDD during the life course, which needs to be considered in future analyses of this data on psychological and familial issues.

## DECLARATION OF INTEREST STATEMENT

None.

## APPENDIX A.

Summary of the questionnaires completed at CoLaus|PsyCoLaus baseline

**Table:**

Name	Version	
	English	French
State–Trait Anxiety Inventory (STAI)	(Spielberger, 1983)	(Spielberger, 1993)
Retrospective Self Report Childhood Inhibition (RSRCI)	(Reznick, Hegeman, Kaufman, Woods, & Jacobs, 1992)	(Tercier et al., 2011)
Dimensions of Temperament Survey Revised (DOTS‐R)	(Windle & Lerner, 1986)	—
Eysenck Personality Questionnaire (EPQ)	(Eysenck & Eysenck, 1975)	—
Type A questionnaire	(Pichot et al., 1977)	—
Sensitivity to Reward (STR)	(Davis, Claridge, & Dransfield, 2003)	—
Parental Bonding Instrument (PBI)	(Parker, Tupling, & Brown, 1979)	(Mohr, Preisig, Fenton, & Ferrero, 1999)
Family Adaptability and Cohesion Scale (FACES III)	(Olson, Portner, & Lavee, 1985)	(Vandeleur, Preisig, Fenton, & Ferrero, 1999)
Dyadic Adjustment Scale (DAS)	(Spanier, 1989)	(Vandeleur, Fenton, Ferrero, & Preisig, 2003)
Family Attitude Scale (FAS‐30)	(Kavanagh et al., 1997)	(Vandeleur, Kavanagh, Favez, Castelao, & Preisig, 2013)
Euronet Problem Resolution Strategy	(Grob & Bodmer, 1996)	(Perrin et al., 2014)
Medical Outcomes Study (MOS) Sleep measure	(Hays, Martin, Sesti, & Spritzer, 2005)	—
